# Public support for healthcare-mediated disclosure of hereditary cancer risk information: Results from a population-based survey in Sweden

**DOI:** 10.1186/s13053-020-00151-0

**Published:** 2020-09-15

**Authors:** Andreas Andersson, Carolina Hawranek, Anna Öfverholm, Hans Ehrencrona, Kalle Grill, Senada Hajdarevic, Beatrice Melin, Emma Tham, Barbro Numan Hellquist, Anna Rosén

**Affiliations:** 1 Department of Radiation Sciences, Umeå University, Umeå, Sweden; 2 Department of Clinical Sciences, University of Gothenburg, Gothenburg, Sweden; 3 Department of Clinical Genetics and Pathology, Laboratory Medicine, Office for Medical Services, Region Skåne, Lund, Sweden; 4 Division of Clinical Genetics, Department of Laboratory Medicine, Lund University, Lund, Sweden; 5 Department of Historical, Philosophical and Religious Studies, Umeå University, Umeå, Sweden; 6 Department of Nursing, Umeå University, Umeå, Sweden; 7 Department of Molecular Medicine and Surgery, Karolinska Institute, Solna, Sweden; 8 Department of Clinical Genetics, Karolinska University Hospital, Solna, Sweden

**Keywords:** Hereditary cancer, Family disclosure, Informing relatives, Healthcare disclosure, Public opinion, Risk information, Cancer prevention, Colorectal cancer

## Abstract

**Background:**

Targeted surveillance of at-risk individuals in families with increased risk of hereditary cancer is an effective prevention strategy if relatives are identified, informed and enrolled in screening programs. Despite the potential benefits, many eligible at-risk relatives remain uninformed of their cancer risk. This study describes the general public’s opinion on disclosure of hereditary colorectal cancer (CRC) risk information, as well as preferences on the source and the mode of information.

**Methods:**

A random sample of the general public was assessed through a Swedish citizen web-panel. Respondents were presented with scenarios of being an at-risk relative in a family that had an estimated increased hereditary risk of CRC; either 10% (moderate) or 70% (high) lifetime risk. A colonoscopy was presented as a preventive measure. Results were analysed to identify significant differences between groups using the Pearson’s chi-square (χ^2^) test.

**Results:**

Of 1800 invited participants, 977 completed the survey (54%). In the moderate and high-risk scenarios, 89.2 and 90.6% respectively, would like to receive information about a potential hereditary risk of CRC (χ2, *p* = .755). The desire to be informed was higher among women (91.5%) than men (87.0%, χ2, *p* = .044). No significant differences were found when comparing different age groups, educational levels, place of residence and having children or not. The preferred source of risk information was a healthcare professional in both moderate and high-risk scenarios (80.1 and 75.5%). However, 18.1 and 20.1% respectively would prefer to be informed by a family member. Assuming that healthcare professionals disclosed the information, the favoured mode of information was letter and phone (38.4 and 33.2%).

**Conclusions:**

In this study a majority of respondents wanted to be informed about a potential hereditary risk of CRC and preferred healthcare professionals to communicate this information. The two presented levels of CRC lifetime risk did not significantly affect the interest in being informed. Our data offer insights into the needs and preferences of the Swedish population, providing a rationale for developing complementary healthcare-assisted communication pathways to realise the full potential of targeted prevention of hereditary CRC.

## Background

Colorectal cancer (CRC) is one of the most common cancers in both sexes, and the second leading cause of cancer death in Sweden [1]. Approximately 3% of CRC cases are attributed to pathogenic variants in mismatch repair genes causing Lynch syndrome [2], resulting in up to 70% lifetime risk of developing CRC [3]. Targeted surveillance in high-risk families has been shown to reduce both cancer morbidity and cancer mortality [3, 4]. Additionally, individuals with no detected pathogenic germline variant, but doubled CRC risk (familial CRC) are recommended colonoscopy surveillance. Regular colonoscopies offered to at-risk individuals have been reported to reduce CRC-related morbidity and mortality by 43–80% and 65–81% respectively [5].

In Sweden and most other countries, the prevailing practice is to encourage the proband (the first individual receiving genetic counselling in a family) to pass on information regarding cancer risk and preventive measures to their at-risk relatives [6]. However, the responsibility to inform at-risk relatives is sometimes burdensome [7]. Several factors have been identified as potential barriers to information spreading. These include conflicts within the family, unwillingness to upset others, selective informing, lack of information, misunderstandings and forgetfulness [8–11].

The success of targeted cancer prevention through surveillance programmes is dependent on effective disclosure of correct information to individuals at risk [12]. Without clear information, individuals are denied the possibility of making an informed decision about predictive testing and potential pursuit of preventive measures. Previous studies have indicated a high interest to undergo genetic testing for hereditary CRC among the public [13, 14]. Despite this, the actual uptake of predictive testing has been reported to vary widely with most studies finding less than half of eligible at-risk relatives being tested [15, 16]. For those at risk of familial CRC, the uptake of surveillance colonoscopy has been reported to be only 34% [5]. Besides the individual benefit of a surveillance programme, the cost-effectiveness of such programmes is directly linked to the amount of identified at-risk relatives who enter surveillance presymptomatically [17, 18].

In this study we explore the general public’s opinion and interest in receiving, and disclosing hereditary CRC risk information. We also investigate preferences for the source and the mode of communicating this information.

## Methods

### Setting

Healthcare expenditure in Sweden is mainly financed by taxes. Regional authorities are responsible for funding and providing healthcare services, while responsibility for overall health policies are managed on a national level [19]. Access to healthcare is heavily subsidised or free for the individual citizen. Patient fees are regulated to a maximum total cost of €122 (SEK 1100) per person for (public) healthcare annually. Nationwide, there are specialised hereditary cancer clinics in six regions, offering genetic counselling, in Sweden called a *family investigation* or *hereditary cancer investigation*. This services include genetic testing or risk assessment based on family history and referrals to surveillance programmes or risk reducing surgery.

### Sample and data collection

Data collection was conducted through a national research infrastructure administered by the Laboratory of Opinion Research (LORE) at the University of Gothenburg in Sweden. Respondents (*n* = 1800), pre-stratified by age, sex and education, were recruited from a random probability-based sample (approximately 9000 individuals) of the general Swedish population. For full details see *Technical report Citizen Panel 31–2018* [20]. Data collection was conducted between the 12th of September and the 7th of October, 2018, during which two reminders were sent to non-responders, 6 and 14 days after the survey was first distributed. Responses with missing data were discarded from analysis (*n* = 13).

### Questionnaire design

The questionnaire was designed and revised through a number of steps. After a literature review, we incorporated qualitative data from a parallel explorative study using focus group discussions (unpublished work, manuscript in writing). Four sessions were conducted with participants recruited by a mix of convenient sampling and snowball samling from different social contexts. The participants (*n* = 15) consisted of 6 men and 9 women, aged 29–64 and level of education ranging from nine-years of primary school to undergratuate degree. We used a semi-structured interview guide with open probing questions and scenario-based questions. Preliminary results on topics raised by participants guided the selection and phrasings of questions in the draft for the survey. The first draft of the questionnaire was reviewed by experts in nursing, clinical genetics, ethics, and oncology. The resulting second draft was tested in a brief pilot study (*n* = 25). Pilot participants consisted of 11 men and 14 women, aged 21–74 and level of education ranging from nine-years of primary school to undergraduate degree. Participant feedback led to editing and rephrasing of questions to improve readability, accessibility and understanding. The final 24-items for this study were administered in Swedish. A translated version is available in Additional file 1.

### Measures

**Fig. 1:**
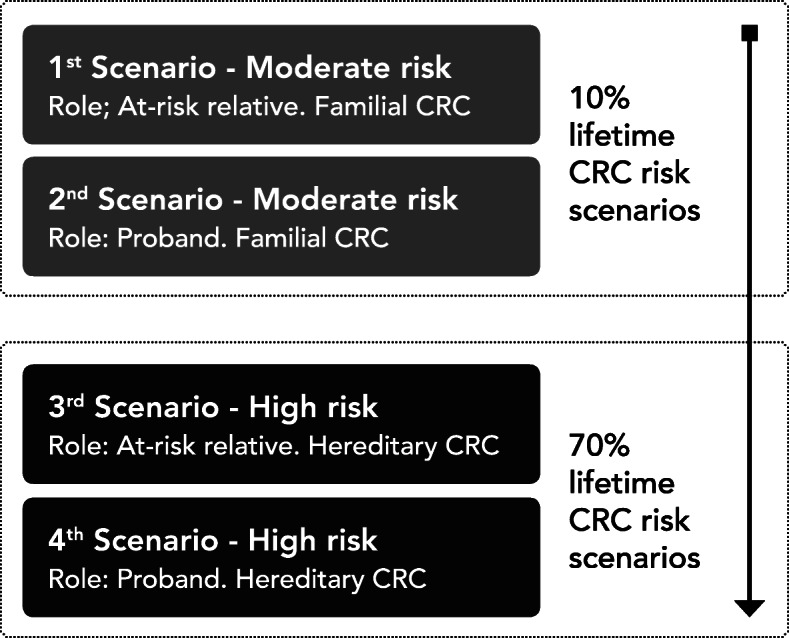
Schematic order of scenarios in the survey. Figure 1 shows the outline of scenarios in the order presented to respondents in the survey. First, the two moderate risk scenarios were presented with accompanying questions from the perspective of an at-risk relative or proband. After this, two new scenarios were presented, this time with a high risk of hereditary CRC, again with questions asked from two alternating perspectives

The questionnaire presented scenarios with adjacent multiple-choice questions requiring one checkbox response per question, or in some items a text answer option. Each scenario also contained an open-ended comment box as a final item. Four scenarios positioned the respondent in a fictional situation of belonging to a family with an increased risk for CRC, focusing on the participant’s opinion on receiving, and passing on, CRC risk information (Fig. 1). Two of the scenarios described a familial cancer situation, where the lifetime risk of CRC was presented as being around 10% (moderate risk). The two other scenarios described a Lynch syndrome situation with hereditary CRC where the lifetime risk was presented to be around 70% (high risk).

Each of the four scenarios consisted of six items. Questions explored whether the respondents themselves would like to be informed about a potential hereditary risk of CRC and if they would like their at-risk relatives to be informed. Also, preferences concerning the source of information (healthcare, relative or other) and mode of information (such as letter, phone, digitally or other) were also explored. Sociodemographic factors, such as age, sex and level of education, were obtained directly from LORE. Other parameters like personal cancer history and place of residence were obtained from additional self-reported demographic questions.

### Statistical analysis

Questionnaire data was compared at the group level, and the distribution of categorical variables was summarized as counts and proportions. Preferences on being informed and disclosing information to a relative were originally captured with 4 predetermined response alternatives. “No, absolutely not” and “No, I don’t think so” was clustered as “No” and the response alternatives “Yes, I think so” and “Yes, absolutely” was clustered as “Yes”. Internal nonresponses are presented in the analysis. Comparisons of proportions were made with the Pearson’s chi-square (χ2) test. Multivariable logistic regression was conducted to control for sex, age, educational level, country of birth, place of residence, having children or not, household status and personal cancer history. All analyses were carried out using the statistical software R, version 3.5.2 [21].. The significance threshold was set at *p* < .05.

### Ethical considerations

Participation in the Citizen Panel was voluntary, and panellists were free to leave the panel at any time. The panellists did not receive any financial incentives for their participation. We provided contact details to the research team in case the content of the questionnaire would induce any cancer worry. The authors only received anonymous survey data and all personal information was stored in encrypted files handled by staff at University of Gothenburg. This study was approved by the Regional Ethical Review Board in Umeå.

## Results

Of 1800 subjects invited, 990 respondents participated. Thirteen responses were excluded due to missing data. Nine hundred seventy-seven respondents completed the survey, and were included in the analysis, resulting in a participation rate of 54%. Table 1 shows a comparison of characteristics in the general Swedish population (aged 18–74), the invited sample, and the study population. Older age, being born in Sweden, higher educational level and having children were associated with a higher response rate, making these groups overrepresented in the sample.

**Table 1:** Distribution of characteristics in the general population, sample and participating respondents

	Subgroup	Population Sweden^a^		Sample		Respondents		Chi-square test^b^
		N	%	N	%	N	%	
Sex	Female	10,720,875	51	890	49	461	47	
	Male	10,422,378	49	910	51	516	53	
	NA	0	0	0	0	0	0	0.198
Age	18–29	4,688,303	22	386	21	131	13	
	30–39	3,776,820	18	334	19	150	15	
	40–49	3,919,526	19	324	18	167	17	
	50–59	3,742,675	18	242	13	158	16	
	60–69	3,408,365	16	304	17	209	21	
	70–74	1,607,564	8	210	12	162	17	
	NA	0	0	0	0	0	0	< 0.01**
Education^c^	Low	13,369,759	61	744	41	392	39	
	Middle	3,127,872	14	538	30	313	32	
	High	4,763,310	22	449	25	266	27	
	NA	514,046	2	69	4	6	1	< 0.01**
Country of birth^d^	Sweden	16,725,884	79	1543	86	899	92	
	Other	4,417,369	21	138	7	68	7	
	NA	0	0	119	7	10	1	< 0.01**
Children^e^	Yes	10,289,146	49	996	55	637	65	
	No	10,811,546	51	721	40	333	34	
	NA	42,561	0	83	5	7	1	< 0.01**
Total	–	21,143,253	–	1800	–	977	–	

Shows the distribution of key characteristics among the Swedish general population (left columns), the invited sample (middle columns) and participating respondents (right colums). Notes about sub-groups presented:

^a^Swedish population data retrieved from publicly available reports by Statistics Sweden (SCB). We used data of the sum total of individuals aged 18–74 years residing in Sweden between the years 2015–2017 as comparison. Population numbers for having children are based on data with “children residing in the household”, in contrast with our respondent data based on the question “do you have children or not?”

^b^Chi-square tests compared population with respondents

^c^Education levels clustered into Low (some elementary or high school education), Middle (post-secondary education < 3 years) or High (3 years of post-secondary education or more)

^d^Self-reported country of birth with response options; Sweden, Europe or Outside Europe

^e^Respondents’ answers to the question; “Do you have children?”

### Opinion on receiving risk information

**Fig. 2:**
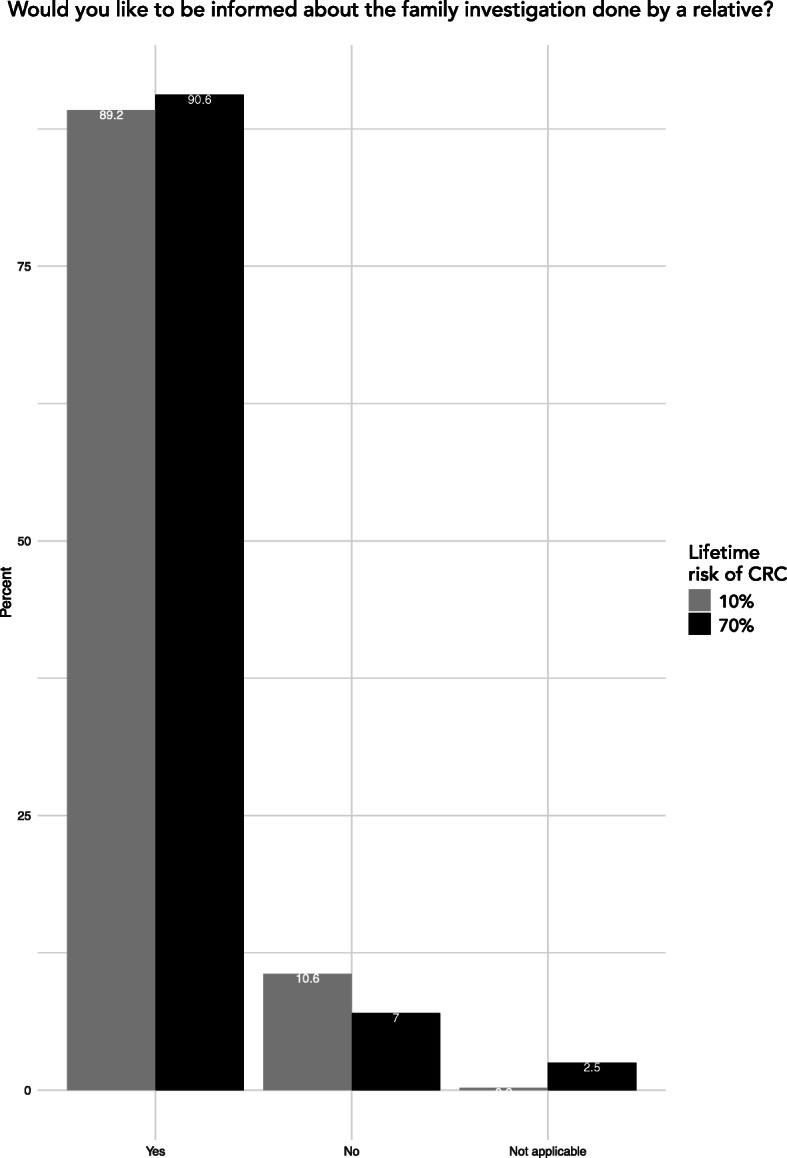
Proportion of responses in the two scenarios of being an at-risk relative. The bars show percentage distribution of respondents reported preferences when answering as an at-risk relative in a family with either moderate (grey bars) or high risk of hereditary CRC (black bars)

The proportion of respondents wishing to be informed of a potential risk of hereditary CRC was 89.2% when answering as an at-risk relative in a family with a moderate risk of CRC (grey bars, Fig. 2). In the scenario where the respondents were presented with a high risk of CRC, 90.6% wished to be informed (black bars, Fig. 2). The two levels of presented cancer risk did not significantly affect the preference to be informed (χ^2^, *p* = .33).

Subgroup analysis showed that a higher proportion of women (91.5%) wanted to be informed about a moderately increased risk as compared to men (87.0%, χ^2^, *p* = .04). No significant difference was seen between groups regarding age, educational level, country of birth, place of residence, having children or not, household status and personal cancer history (Table 2). We performed the same subgroup analysis for the scenario of belonging to a family with high risk of CRC which showed similar results (data not shown). In the logistic regression adjusting for age, educational level, country of birth, place of residence, having children or not, household status and personal cancer history, differences between sexes was unsignificant (*p* = .0594).

**Table 2:** Respondents’ preferences on the disclosure of risk information in the scenario of moderate CRC risk

Group	Subgroup		***As relative:*** *Want to receive information about a potential hereditary CRC risk*			Chi-square test^a^	***As proband:*** *Want relatives to be informed about their potential hereditary CRC risk*			Chi-square test^a^
			**Yes**	**No**	**NA**	***p*-value**	**Yes**	**No**	**NA**	**p-value**
**Sex**	Women	N	422	39	0		430	28	3	
		%	91.5	8.5	0		93.3	6.1	0.7	
	Men	N	449	65	2		451	52	13	
		%	87.0	12.6	0.4		87.4	10.1	2.5	
						0.04*				0.02*
**Age**	18–39	N	253	26	2		255	17	9	
		%	90.0	9.3	0.7		90.7	6.0	3.2	
	40–59	N	291	34	0		291	29	5	
		%	89.5	10.5	0		89.5	8.9	1.5	
	60–74	N	327	44	0		335	34	2	
		%	88.1	11.9	0		90.3	9.2	0.5	
						0.58				0.34
**Education**^b^	Low	N	348	43	1		349	33	10	
		%	88.8	11	0.3		89.0	8.4	2.6	
	Middle	N	278	34	1		280	28	5	
		%	88.8	10.9	0.3		89.5	8.9	1.6	
	High	N	240	26	0		246	19	1	
		%	90.2	9.8	0		92.5	7.1	0.4	
						0.87				0.69
**Country of birth**^c^	Sweden	N	806	91	2		811	75	13	
		%	89.7	10.1	0.2		90.2	8.3	1.4	
	Other	N	57	11	0		61	5	2	
		%	83.8	16.2	0		89.7	7.4	2.9	
						0.18				0.98
**Place of residence**	City	N	674	81	1		686	59	11	
		%	89.2	10.7	0.1		90.7	7.8	1.4	
	Rural	N	194	22	1		192	21	4	
		%	89.4	10.1	0.5		88.5	9.7	1.8	
						0.92				0.45
**Children**^d^	Yes	N	565	71	1		573	55	9	
		%	88.7	11.1	0.2		90.0	8.6	1.4	
	No	N	300	32	1		302	25	6	
		%	90.1	9.6	0.3		90.7	7.5	1.8	
						0.53				0.64
**Single household**^e^	Yes	N	150	19	1		152	14	4	
		%	88.2	11.2	0.6		89.4	8.2	2.4	
	No	N	705	83	1		714	65	10	
		%	89.4	10.5	0.1		90.5	8.2	1.3	
						0.89				1.00
**Personal cancer history**^f^	Yes	N	76	7	0		80	3	0	
		%	91.6	8.4	0		96.4	3.6	0	
	No	N	773	90	1		783	76	5	
		%	89.5	10.4	0.1		90.6	8.8	0.6	
						0.70				0.13
**Total**		**N**	871	104	2		881	80	16	
		**%**	89.2	10.6	0.2		90.2	8.2	1.6	

Shows respondents’ detailed characteristics versus reported preferences on the disclosure of risk information when answering as a proband, or an at-risk relative, belonging to a family with a moderately increased hereditary risk of CRC (10% lifetime risk)

^a^Chi-square tests compared proportions in the different groups

^b^Education levels clustered into Low (some elementary or high school education), Middle (post-secondary education < 3 years) or High (3 years of post-secondary education or more)

^c^Self-reported country of birth with response options; Sweden, Europe or Outside Europe

^d^As per response to the question; “Do you have children?”

^e^Household status “single” corresponds to residing alone currently

^f^Having a current or past cancer diagnosis classified as yes, no previous or known cancer classified as no.

### Opinions on disclosure of risk information

**Fig. 3:**
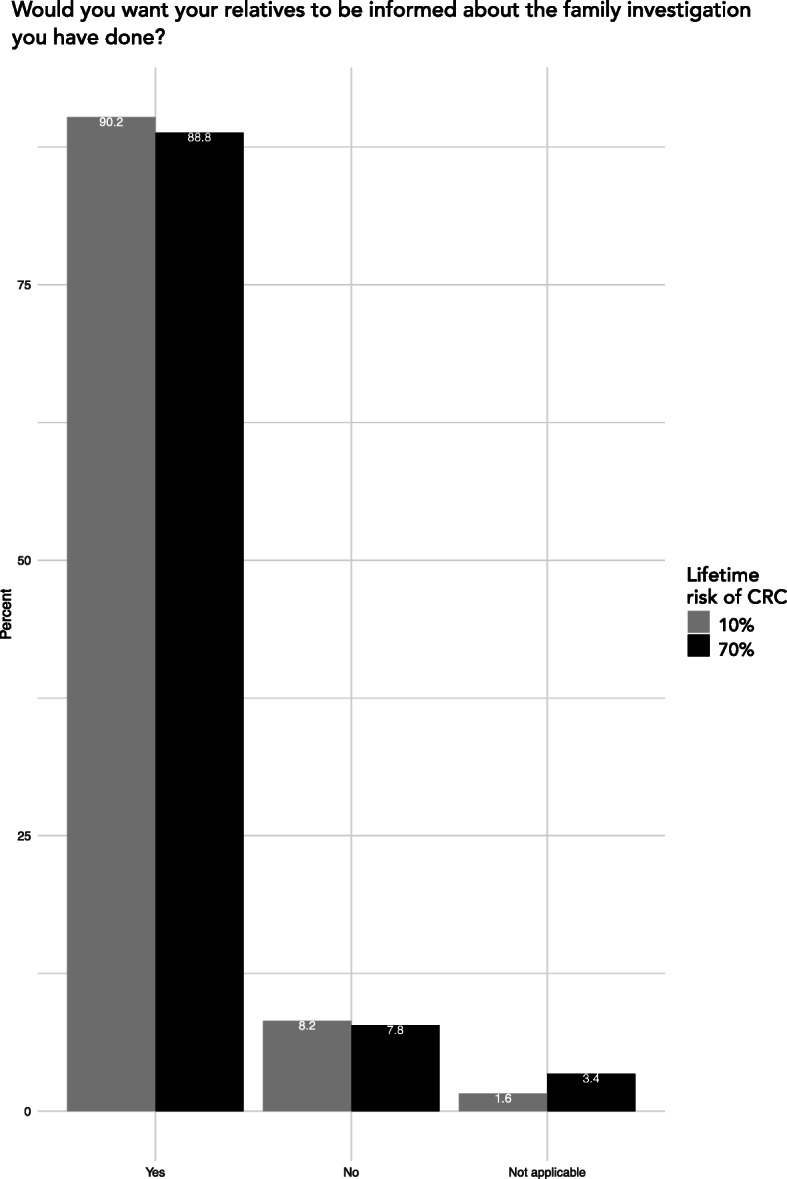
Proportion of responses in the two scenarios of being a proband. The bars show the percentage disctribution of respondents’ reported preferences when answering as a proband in a family with either moderate lifetime risk (grey bars) or high life time risk of CRC (black bars)

The proportion of respondents wanting their relatives to be informed about a potential moderate risk of CRC was 90.2% (grey bars, Fig. 3). In the scenario with a high risk of CRC, 88.8% of respondent wanted their relatives to be informed (black bars, Fig. 3). The two risk levels did not significantly affect the preference to have their relatives informed (χ^2^, *p* = .755). Subgroup analysis detected a difference between sexes in the moderate risk scenario; 93.3% of women wanted their relatives to be informed, compared to 87.4% of men (χ^2^ *p* = .024, Table 2). No significant difference was seen between groups regarding age, educational level, country of birth, place of residence, having children or not, household status and personal cancer history. We performed the same subgroup analysis for the scenario with high risk of CRC which showed similar results (data not shown). The gender difference in desire to inform relatives remained significant when we adjusted for age, educational level, country of birth, place of residence, having children or not, household status and personal cancer history in a multivariable logistic regression (*p* = .0260).

### Preferred source of cancer risk information

**Fig. 4:**
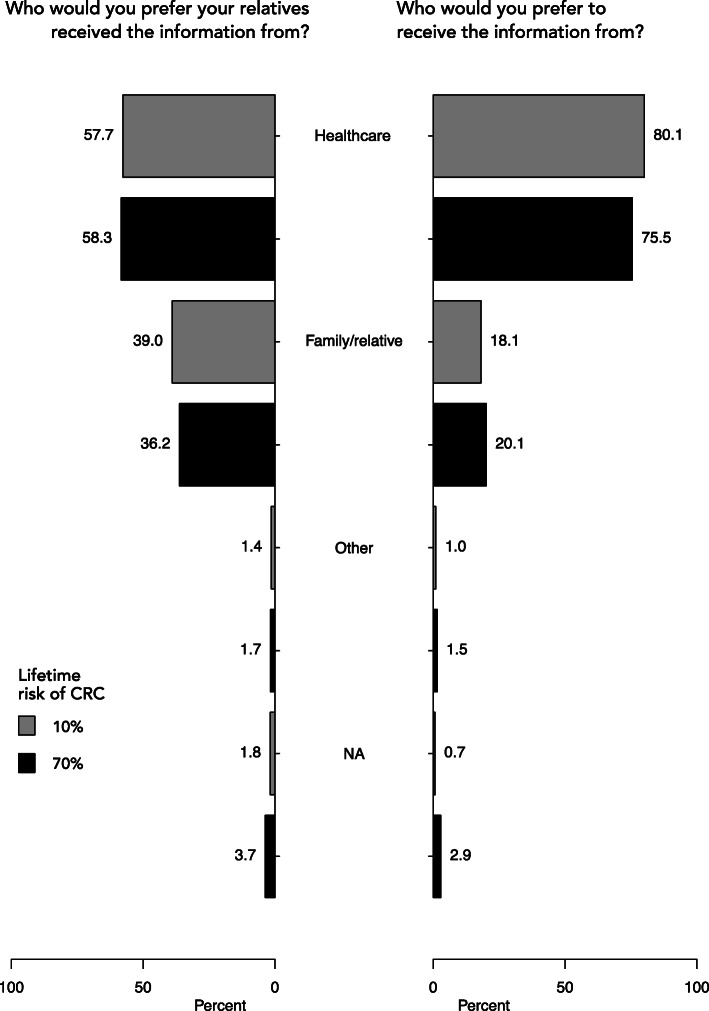
Proportion of responses on preferred information source. The bars show percentages of responents’ preferences on whom they would want their relatives received risk information from, and who they themselves would prefer to receive risk information from. Grey bars show responses from the moderate lifetime CRC risk scenarios and black bars show reponses from the high lifetime CRC risk scenarios

When answering as an at-risk relative in a family with moderate risk, 80.1% of respondents preferred healthcare-mediated disclosure of information. 18.1% would rather receive the information from a family member (grey bars, left side, Fig. 4). When answering as a proband, 57.7% selected healthcare as the preferred mediator of risk information. 39.0% preferred to disclose risk information by themselves (grey bars, right side, Fig. 4).

The two high-risk scenarios revealed a similar distribution of preferences regarding the source of information (black bars, Fig. 4). We found no significant differences regarding the preferred source of information between different sexes, age groups, educational level, place of residence, having children or not, household status and personal cancer history (data not shown).

### Preferred mode of communication

**Fig. 5:**
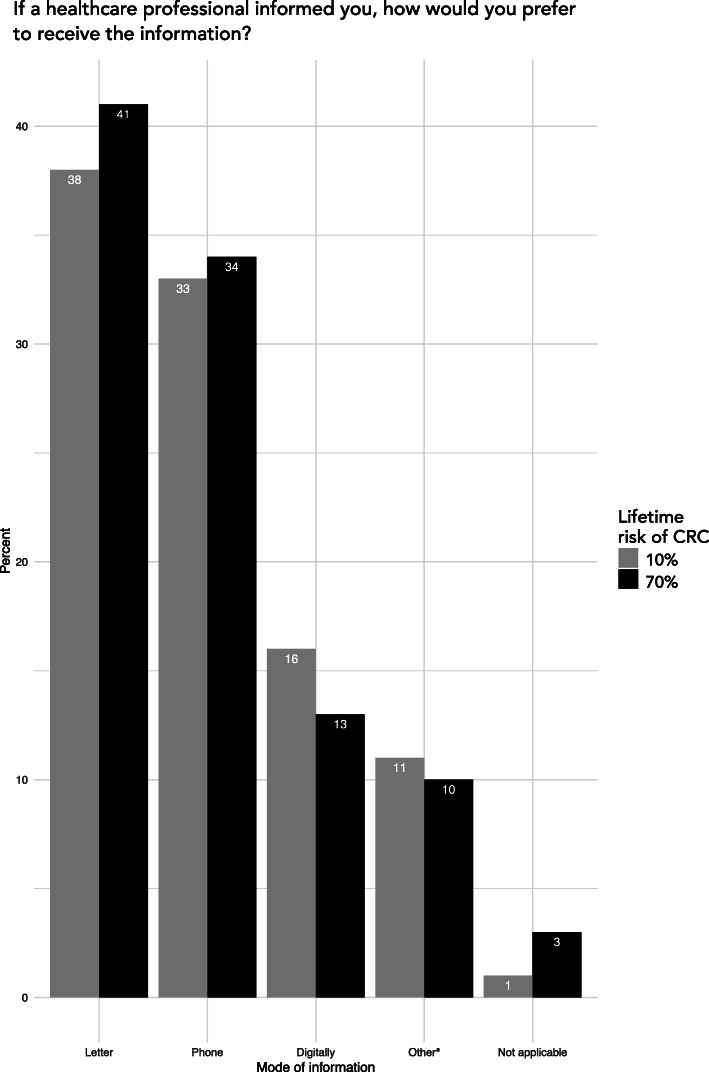
Preferred mode of risk information (in healthcare-assisted scenario). Bars show percentage distribution of respondents’ preferred way to receive risk information given the scenario that disclosure was handled by a healthcare professional. Pre-selected response options included “letter”, “phone”, “digitally” or “other”. ^a^ The response alternative “other” was an open-ended option where 106 of 111 respondents in some way described a personal meeting or counselling session as the preferred mode of information

If a healthcare professional would deliver information on a moderate risk of CRC, respondents would prefer to receive information through a letter (38.4%) or a telephone call (33.2%, Fig. 5). The response alternative “other” was preferred among 11.4%. When specified in the comments, a majority (106 out of 111) expressed that they would instead prefer some sort of face-to-face meeting or consultation with a healthcare professional. The distribution of preferences was similar in the high-risk scenario. If a relative would deliver the information about a moderate risk of CRC in the family, the preferred mode of communication was a personal meeting (58.2%), followed by telephone (24.0%), letter (5.9%), digitally (6.7%), other (1.1%), NA (4.0%).

## Discussion

To explore public opinion on the disclosure of genetic risk information we used scenarios presenting respondents with hypothetical scenarios of belonging to a family with two different levels of increased risk of CRC.

### People want to be informed

A majority of the respondents in our moderate and high-risk scenarios would want to receive information about a potential risk of CRC. In a recent Danish study, Petersen et al. reported that 82% in a population survey wished to obtain personal CRC risk information [22]. In a British survey, 91% wanted to be contacted about a “preventable and fatal disease” [23]. Thus, a growing body of evidence [22–26], including this study, suggests that a majority of the public has a strong preference to be informed about possible hereditary cancer risk, and some even advocate for breaching of confidentiality if the proband does not consent to disclosure [23, 25].

In our study, women expressed more interest in receiving information than men, but the association was only significant in univariate analysis and not when adjusted for other factors. No other factor was found to significantly affect preferences, including the level of disease risk (moderate or high lifetime risk of CRC). Previous studies have shown varying results regarding gender-specific preferences. Wolff et al. found gender to have a small but significant effect on the willingness to be informed or not [25]. Overall, the desire to be informed, and to disclose risk information, seem to be primarily modulated by context factors such as the treatability of disease [23, 25], seriousness of disease [23], accuracy of tests and privacy issues [14], rather than characteristics of respondents. This study used specific CRC-scenarios, thus keeping several of the determining factors above constant. For instance treatability and seriousness of disease was the same throughout all scenarios, allowing us to assess factors such as risk levels and privacy preferences in detail within this context.

### People want their relatives to be informed

Intention to disclose risk information to relatives was high in our study; 90.2% of respondents wanted their relatives to be informed in the moderate risk scenario, and 88.8% in the high-risk scenario. Nevertheless, research has shown that in real life family-mediated disclosure of risk information is not very effective [27]. Daly et al. showed that 80% of probands in their study reported disclosure to at least one first-degree relative [9]. But when researchers contacted relatives for follow-up, 22% of the relatives said that they had in fact never received any information. Roshanai et al. also report on follow-up of family-disclosure; 73% of probands reported informing at least one at-risk relative. But when probands were asked for permission to contact relatives, only about half provided contact information, and half of the contacted relatives accepted to participate in the study [28]. Of those who agreed to participate, around half intended to seek genetic counselling themselves.

The strong preferences to inform relatives can be seen as a sign of good intentions. However, as previous literature show, family-mediated disclosure can lead to inaccurate information or misunderstandings. In an observational study, Jacobs et al. showed that relatives who receive information from the proband alone recalled significantly less accurate information than relatives who received information from several sources, such as from genetic clinics [29]. Therefore, while it is clear that people generally want to be informed, and want their relatives to be informed, the question of designing a supportive and effective practice to reach eligible at-risk relatives still remain to be answered.

### Healthcare the preferred source of information

If another family member had undergone genetic counselling, revealing a potential hereditary risk of CRC for relatives, the majority of respondents in our sample would prefer healthcare professionals to communicate the risk information (answering as the relative). In Denmark, a recent study similarly showed that 66% of respondents preferred a letter from healthcare over information from a family member [22]. In our study, the proportion of respondents favoring healthcare-mediated disclosure was even higher (80.1 and 75.5%, moderate and high risk respectively).

Interestingly, healthcare-mediated dislosure was also the most attractive option when respondents answered in the role of being the proband passing on information. However, here the numbers are lower (57.7 and 58.3%), indicating that these two opposing roles (at-risk relative vs. proband) do affect people’s preferences on how the information should be mediated. The prevailing view in the literature is that probands favour family-mediated risk disclosure, but desire active support from healthcare professionals, as described in a recent review by van den Heuvel et al. [30]. Our data including both perspectives (of being a relative and a proband), although in a ficional situation, supports the notion that acceptance for health care assisted risk disclosure seems to be high. It remains to be seen if these contradicting results reflect a current shift in the public opinion or differences between nationalities or cultural contexts.

There is an ongoing debate over whether healthcare professionals should take on a more proactive role in the disclosure of genetic information to at-risk relatives [30–32]. Studies which have evaluated a more proactive approach with information letters sent directly to at-risk relatives have reported a significant increase, in some cases doubling, in the numbers of relatives who seek genetic counselling and testing [33, 34].

In a few countries, healthcare professionals are expected to act proactively when needed. Direct contact between healthcare professionals and at-risk relatives is, for example, sanctioned by the legal system in Australia in special cases [35]. In France, the legal changes enacted between 2011 and 2013 now make probands legally required to disclose relevant information if a relative is at risk for a hereditary disease. The counselee is given two options; either to disclose positive test results themselves, or to let healthcare professionals contact their at-risk relatives in their place [36].

### Preferred mode of communication

The majority of respondents chose letter or phone as preferred mode of communication if information would be delivered from healthcare professionals. The respondents who selected the open-ended option “other” (10.3 and 11.4% in the two risk scenarios) commented that they would prefer a personal consultation or meeting. In this question, we purposefully chose to exclude a pre-defined response option with “a personal meeting” since in clinical practice, personal counselling would still require a first contact (by letter or phone) in which the reason for the consultation must be given. Previous studies show that “a personal meeting” is reported as the most attractive option if such a response alternative is offered [37].

Some research on direct letters to relatives has recently been published [22, 38]. In France, Zordan et al. evaluated templates for letters sent directly from healthcare to previously uncontacted at-risk relatives. They conclude that despite initial feelings of anxiety, the understanding and reported motivation to seek counselling in individuals who were successfully contacted was high. However, the authors call for more research on follow-up and quantification of actual testing uptake following such direct contact approaches [38]. In Denmark, Petersen et al. report that unsolicited information letters were supported by 82% of the general population, and 78% of at-risk relatives. 90% of family members preferred a letter to no information and 66% preferred information from healthcare over family-mediated information [22].

### Methodological considerations

A number of limitations need to be considered when interpreting this data. We used an electronic survey format which could affect the selection of respondents. Individuals with limited computer literacy, limited Swedish language skills or lower health literacy may be underrepresented. Research on online questionnaire respondents however does suggest that respondents are comparable with those responding to traditional data collection methods e.g. postal questionnaires [39–41]. To collect data representative of the Swedish general population, we recruited respondents from a probability-based sample and pre-stratified respondents by age, sex and educational level. The higher response rate in some subgroups made some characteristics (like higher age and education) more common in our sample (Table 1). Interpretations concerning individuals with lower education, younger age and those not born in Sweden should therefore be made with caution. Non-response may also have contributed to selection bias, but since our survey was part of a series of questionnaires it is unlikely that non-respondents were affected by the topic investigated. The response rate in our subsample of the citizen panel (54%) was close to the overall response rate of the full panel (57%) [20].

Contextual aspects specific for this population, such as social culture, family dynamics and high trust in the healthcare system, limit the generalization of our results to other countries. Our study was performed in a Scandinavian country with heavily subsidised, general access to public healthcare. Opinions in countries with larger out-of-pocket expenditure and differing societal values may differ. For instance, a population-based study in the US, on the related topic “interest in genetic testing for hereditary risk of CRC” reported personal cost and privacy as the most important determinants of respondents willingness to undergo genetic testing [14]. The decision to undergo a genetic test is a closely related, but not an identical concept to the one investigated in our study. A decision about testing will in practice first require risk information disclosure by someone. Public opinion on genetic testing can however be suspected to be closely associated with public opinion on risk information disclosure.

Whether our results are transferable to a real-life setting also depend on the differences between being exposed to a hypothetical research situation or dealing with the equivalent real-life situation. Wolff et al. reported a disparity between the general public and patients who had received genetic counselling concerning hereditary cancer in their desire to be informed about the existence of hereditary conditions within their family. Both groups wanted to be informed, but patients were more positive towards being informed [24].

### Implications

As genetic testing becomes more frequent in clinical practice, the number of actionable solicited and unsolicited findings concerning people other than the primary patient will increase. It is therefore likely that healthcare professionals will more often face ethical and practical dilemmas regarding the disclosure of hereditary risk information in the coming years. A better understanding of the evolving opinions among the public, and thus potential future patients, is a good foundation for further work in this field.

Since the cost-effectiveness of targeted surveillance programmes depends on reaching and enrolling individuals at-risk [17], future research could explore ways to implement a more collaborative approach on risk information disclosure. The data presented in this study add to the body of evidence showing high acceptability for healthcare-assisted disclosure pathways [22, 23, 32]. However, significant clinical challenges remain, such as the limited counselling and testing resources, difficulties in obtaining contact information to relatives and managing structured follow-up. This study is part of a wider explorative research project, and results have guided the design of an RCT protocol (ClinicalTrials.gov Identifier: NCT04197856). The resulting national clinical study is currently underway, recruiting patients at four hereditary cancer clinics in Sweden, and will compare effectiveness between current clinical praxis and a healthcare-assisted option offering direct contact by letter from healthcare provider to at-risk relatives.

## Conclusions

In this study, we have shown that a majority of respondent in a Swedish population-based sample would like to receive and disclose hereditary cancer risk information. Moreover, a majority would prefer healthcare-mediated disclosure over family-mediated when confronted with several hypothetical disclosure scenarios on genetic CRC risk. When choosing between different modes of contact, a letter was the most favoured format, closely followed by a telephone call.

Considering the benefits of early cancer prevention, the unsatisfactory results with family-mediated disclosure, and the emerging evidence on public opinion and patients’ preferences, we believe there is an imperative to identify a feasible and acceptable praxis for healthcare-assisted disclosure of genetic information to at-risk relatives.

## Supplementary information

**Additional file 1.** Questionnaire on hereditary cancer risk disclosure (LORE, citizen panel, wave 31, block 5, q96-q127). This additional file contains a complete transcript of the survey questions used to generate the data for this article. The transcript is an English translation of the original Swedish questionnaire.

## Data Availability

The datasets used and/or analysed during the current study are available from the corresponding author.

## Abbreviations

CRC: Colorectal Cancer
LORE: Laboratory of Opinion Research (at Gothenburg University)
NA: Not applicable
