# Supplementary material for: Public support for healthcare-mediated disclosure of hereditary cancer risk information: Results from a population-based survey in Sweden
Source: Hered Cancer Clin Pract. 2020 Sep 15;18:18. doi: 10.1186/s13053-020-00151-0 (PMC7493346; doi:10.1186/s13053-020-00151-0)
Supplement: Supplementary file 1 — Additional file 1. Questionnaire on hereditary cancer risk disclosure (LORE, citizen panel, wave 31, block 5, q96-q127). This additional file contains a complete transcript of the survey questions used to generate the data for this article. The transcript is an English translation of the original Swedish questionnaire. [file 13053_2020_151_MOESM1_ESM.pdf]

Questionnaire on hereditary cancer risk disclosure (lore, citizen panel, wave 31, block 5, q96-q127).
Translation from Swedish by Carolina Hawranek.

Article title: *Public support for healthcare-mediated disclosure of hereditary cancer risk information: Results from a population-based survey in Sweden*

Journal name: *Hereditary Cancer in Clinical Practice*

Author names: *Andreas Andersson, Carolina Hawranek, Anna Öfverholm, Hans Ehrencrona, Kalle Grill, Senada Hajdarevic, Beatrice Melin, Emma Tham, Barbro Numan Hellquist), Anna Rosén*

Corresponding author: *Carolina Hawranek, Department of Radiation Sciences, Umeå University, Carolina.hawranek@umu.se, +46768934504*

---

q96 The following section concerns your thoughts on how hereditary cancer risk information should be handled. In some families there is an increased risk of cancer. Affected relatives can be offered health checks in order to early detect and remove early stages of cancer. The chances of being cured increase greatly if the cancer is discovered early on.

We would like you to imagine being part of six scenarios and answer the accompanying questions. The scenarios are all examples of situations that arise at cancer genetic units in Swedish clinical practice.

This study is a collaboration between Gothenburg University and Umeå University with the aim to improve care at cancer genetic units.

Principal investigator is Anna Rosén, MD at Umeå University.

q98 **Scenario 1.** Your relative Kit has initiated a family investigation at a cancer genetic unit. The investigation shows that several individuals in your family may have a doubled risk of developing colorectal cancer sometime during their life (around 10 percent lifetime risk compared to average 5 percent). Relatives at risk can be offered colonoscopies every fifth year to early detect, or remove, early stages of cancer.

q99 Would you like to be informed about the family investigation done by Kit?

- O No, absolutely not (1)
- O No, I don’t think so (2)
- O Yes, I think so (3)
- O Yes, absolutely (4)

q100 Who would you prefer to receive the information about Kit’s investigation from?

- A relative (1)
- A health care professional (2)
- Other: (3) ____

q101 If a relative informed you, how would you prefer to receive the information?

- By video call (1)
- By letter (2)
- By telephone call (3)
- By email (4)
- By text message (5)
- In a personal meeting (6)
- Other: (7) ____

q102 If a health care professional informed you, how would you prefer to receive the information?

- By video call (1)
- By letter (2)
- By telephone call (3)
- By email (4)
- By text message (5)
- By logging in at ”Mina vårdkontakter”, 1177 Vårdguiden* (6)
- Other: (7) ____

*a secure personal online portal for public health care access and communications in Sweden

q103 Besides information about Kit's investigation, would you also like to find out if you are one of those with an increased risk?

- No, Absolutely not (1)
- No, I don't think so (2)
- Yes, I think so (3)
- Yes, absolutely (4)

q104 If you have any comments on Scenario 1 you are welcome to add them here:

____
____
____
____
____

q106 **Scenario 2.** You have initiated a family investigation at a cancer genetic unit. The investigation shows that several individuals in your family may have a doubled risk of developing colorectal cancer (around 10 percent lifetime risk compared to average 5 percent). Affected individuals can be offered colonoscopies every fifth year to early detect, or remove, early stages of cancer.

q107 Would you want your relatives to be informed about the family investigation you have done?

- No, Absolutely not (1)
- No, I don't think so (2)
- Yes, I think so (3)
- Yes, absolutely (4)

q108 Who would you prefer your relatives received information about your investigation from?

- Yourself (1)
- A health care professional (2)
- Other: (3) ____

q109 If you informed your relatives, how would you prefer to deliver the information?

- By video call (1)
- By letter (2)
- By telephone call (3)
- By email (4)
- By text message (5)
- In a personal meeting (6)
- Other: (7) ____

q110 If a health care professional informed your relatives, how would you prefer they received the information?

- By video call (1)
- By letter (2)
- By telephone call (3)
- By email (4)
- By text message (5)
- By logging in at ”Mina vårdkontakter”, 1177 Vårdguiden* (6)
- Other: (7) ____

*a secure personal online portal for public health care access and communications in Sweden

q111 Besides information about your investigation, would you also like your relatives to find out if they were one of those with an increased risk?

- No, absolutely not (1)
- No, I don’t think so (2)
- Yes, I think so (3)

- Yes, absolutely (4)

q112 If you have any comments on Scenario 2 you are welcome to add them here:
____
____
____
____
____

q114 **Scenario 3.** Your relative Eli has initiated a family investigation showing that Eli has a genetic variant associated with a highly increased lifetime risk of developing colorectal cancer (around 70 percent risk compared to average 5 percent). Since Eli is a carrier of the variant, other individuals in his family may also be carriers. A blood sample can show if you are a carrier or not. Carriers can be offered colonoscopies ever second year to early detect and remove early stages of cancer. If one is a non-carrier there is no increased risk of disease.

q115 Would you like to be informed about the family investigation done by Eli?

- No, absolutely not (1)
- No, I don't think so (2)
- Yes, I think so (3)
- Yes, absolutely (4)

q116 Who would you prefer to receive the information about Eli's investigation from?

- A relative (1)
- A health care professional (2)
- Other: (3) ____

q117 If a relative informed you, how would you prefer to receive the information?

- By video call (1)
- By letter (2)
- By telephone call (3)

- By email (4)
- By text message (5)
- In a personal meeting (6)
- Other: (7) ____

q118 If a health care professional informed you, how would you prefer to receive the information?

- By video call (1)
- By letter (2)
- By telephone call (3)
- By email (4)
- By text message (5)
- By logging in at ”Mina vårdkontakter”, 1177 Vårdguiden* (6)
- Other: (7) ____

*a secure personal online portal for public health care access and communications in Sweden

q119 You are offered to take a blood sample to determine whether you carry the genetic variant. Would you want to complete the genetic test?

- No, absolutely not (1)
- No, I don’t think so (2)
- Yes, I think so (3)
- Yes, absolutely (4)

q120 If you have any comments on Scenario 3 you are welcome to add them here:

____
____
____
____

q122 **Scenario 4.** You have initiated a family investigation showing that you have a genetic variant associated with a highly increased lifetime risk of developing colorectal cancer (around 70 percent risk compared to average 5 percent). Since you are a carrier, other individuals in your family may also be carriers. On the other hand, non-carriers have no increased risk of disease. Carriers can be offered colonoscopies every second year to early detect and remove early stages of cancer.

q123 Would you want your relatives to be informed about the investigation you have done?

- O No, absolutely not (1)
- O No, I don't think so (2)
- O Yes, I think so (3)
- O Yes, absolutely (4)

q124 Who would you prefer your relatives received information about your investigation from?

- O A relative (1)
- O A health care professional (2)
- O Other: (3) ____

q125 If you informed your relatives, how would you prefer to deliver the information?

- O By video call (1)
- O By letter (2)
- O By telephone call (3)
- O By email (4)
- O By text message (5)
- O In a personal meeting (6)
- O Other: (7) ____

q126 If a health care professional informed your relatives, how would you prefer they received the information?

- By video call (1)
- By letter (2)
- By telephone call (3)
- By email (4)
- By text message (5)
- By logging in at ”Mina vårdkontakter”, 1177 Vårdguiden* (6)
- Other: (7) ____

*a secure personal online portal for public health care access and communications in Sweden

q127 Your relatives are offered to take a blood sample in order to determine whether they carry the altered gene. Would you want them to complete the genetic test?

- No, Absolutely not (1)
- No, I don’t think so (2)
- Yes, I think so (3)
- Yes, absolutely (4)

q128 If you have any comments on Scenario 4 you are welcome to add them here:
____
____
____
____

<End of section>
